# Completion of the Icatibant Outcome Survey and What We Learned

**DOI:** 10.1111/cea.70271

**Published:** 2026-03-10

**Authors:** Laurence Bouillet, Werner Aberer, Teresa Caballero, Anete S. Grumach, Hilary J. Longhurst, Alissa Dangel, Natalie Khutoryansky, Irmgard Andresen, Andrea Zanichelli

**Affiliations:** ^1^ National Reference Centre for Angioedema, Internal Medicine Department Grenoble University Hospital Grenoble France; ^2^ Department of Dermatology and Venereology Medical University of Graz Austria; ^3^ National Reference Center (CSUR) for Hereditary Angioedema, Department of Allergy Hospital Universitario La Paz, Hospital La Paz Institute for Health Research (IdiPAZ), Biomedical Research Network on Rare Diseases (CIBERER, U754) Madrid Spain; ^4^ Clinical Immunology, Faculdade de Medicina Centro Universitario Faculdade de Medicina ABC (CEUFMABC) Santo Andre Brazil; ^5^ Department of Medicine University of Auckland and Auckland City Hospital Auckland New Zealand; ^6^ Takeda Pharmaceuticals USA, Inc. Cambridge Massachusetts USA; ^7^ Takeda Development Center Americas, Inc. Lexington Massachusetts USA; ^8^ Takeda Pharmaceuticals International AG Zurich Switzerland; ^9^ Operative Unit of Medicine, Angioedema Center, IRCCS Policlinico San Donato Milan Italy; ^10^ Department of Biomedical Sciences for Health University of Milan Milan Italy

**Keywords:** clinical outcomes, hereditary angioedema, icatibant, long‐term safety

## Abstract

- The Icatibant Outcome Survey observational registry began in 2009 to monitor the safety and effectiveness of icatibant.
- Fifteen years of icatibant use support effectiveness in hereditary angioedema, with no new safety signals.

To the Editor,

The Icatibant Outcome Survey (IOS; NCT01034969) was a prospective, international, observational registry that documented clinical outcomes from adult and paediatric patients with hereditary angioedema (HAE) due to C1 inhibitor defects/dysfunction (HAE‐C1INH). Patients with other types of angioedema, such as HAE with normal C1 inhibitor function (HAE‐nC1INH), were also included. The primary objective for icatibant was to monitor safety during long‐term treatment, with a focus on cardiac ischaemia event frequency in predisposed patients, generalised reactions and effects on sexual maturation in pubertal adolescents. Secondary objectives included treatment effectiveness. The registry was conducted according to the Declaration of Helsinki and International Conference on Harmonisation Good Clinical Practice Guidelines. Local ethics committee approval and written informed consent were obtained [1]. Prior publications have described the IOS registry design in detail [1, 2, 3, 4, 5] and 10‐year data were recently reviewed [6]. Here we summarise 15 years of follow‐up of icatibant‐treated patients from the IOS registry, from initiation through database lock (July 10, 2009–July 15, 2024).

Of the 1624 patients who provided consent, 1022 from 13 countries received ≥ 1 icatibant dose and were included in the icatibant safety set (ISS; 820 with HAE‐C1INH, 99 with HAE‐nC1INH and 103 with other diagnoses; Figure 1a). There were 997 treatment‐emergent adverse events (TEAEs) reported in 368 of 1022 (36.0%) patients; 28 TEAEs (2.8%) reported by 11 patients (1.1%) were possibly related to icatibant, and 70 TEAEs (7.0%) reported by 32 patients (3.1%) were probably related. The most frequently reported icatibant‐related TEAEs were injection‐site erythema (20 events), ineffective treatment (14 events) and application‐site erythema (8 events). Seventeen deaths occurred among patients in the ISS, all unrelated to icatibant. Overall, the number of patients with a completed follow‐up case‐report form (*n* = 887) who experienced generalised reactions during or after treatment with icatibant was low (hypotension [*n* = 7 patients], swelling of mucous membranes [*n* = 14], bronchoconstriction [*n* = 3], or aggravation of pain [*n* = 17]). No further details about the generalised reactions, such as timings relative to icatibant injections, relatedness or resolution, were captured in the database.

**FIGURE 1:**
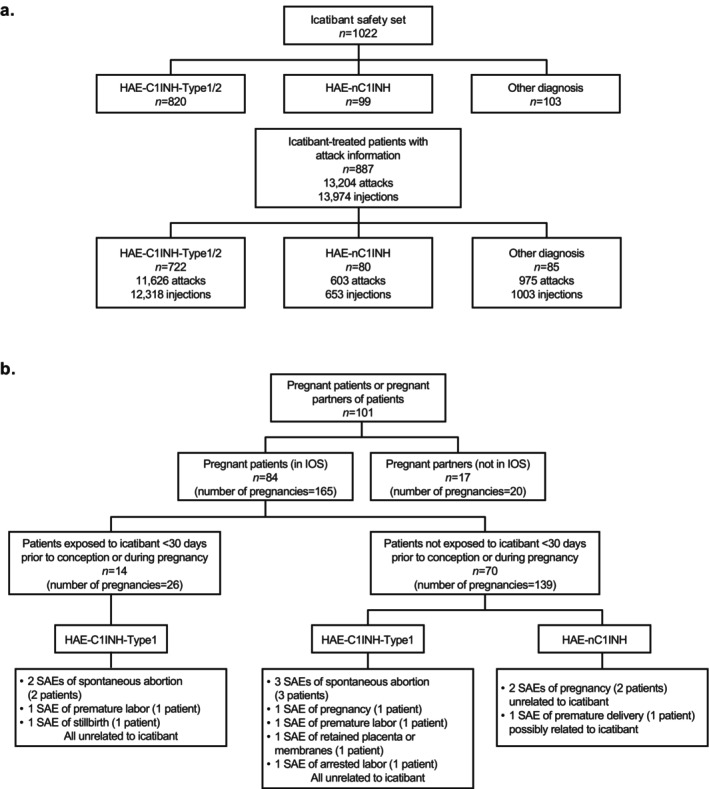
(a) Icatibant safety set and icatibant‐treated patients with a completed follow‐up form showing attack information; (b) pregnancies occurring during the Icatibant Outcome Survey (IOS) registry (July 10, 2009–July 15 2024; icatibant safety set). HAE‐C1INH‐Type1/2, hereditary angioedema due to C1 inhibitor deficiency; HAE‐nC1INH, hereditary angioedema with normal C1 inhibitor levels; n, number of patients; SAE, serious adverse event.

Predisposition and occurrence of cardiac and cerebral ischaemic events were monitored due to the role of bradykinin in ischaemia and previous research in animal models suggesting poorer outcomes with myocardial ischaemia/infarction after icatibant use [7]. Among 1022 patients in the ISS, 151 (14.8%) reported a medical history of cardiac/cardiovascular events, and 7 (0.7%) reported a history of cerebrovascular events. In 887 patients reporting 13,204 HAE attacks (Figure 1a), 16 attacks in 3 patients had symptoms suggestive of cardiac ischaemia during an attack; one of these patients was among those with a medical history of cardiac/cardiovascular events. Symptoms suggestive of cerebral ischaemia were reported in 4 patients for 5 attacks; none of these patients had a history of cerebrovascular events. As symptoms ‘suggestive of cerebral ischemia’ were not recorded as TEAEs by investigators, TEAEs were examined separately. There were 5 cerebral ischaemic TEAEs reported in 5 patients, all considered unrelated to icatibant and none occurred in a patient with a cerebrovascular medical history.

Pregnancy events were also monitored. There were 165 pregnancies among 84 female patients in the ISS. Of the 26 pregnancies in 14 patients with reported exposure to icatibant < 30 days before conception or during pregnancy, all adverse pregnancy outcomes reported were considered unrelated to icatibant (Figure 1b). Since specific details about pregnancies, childbirth and neonates are handled by obstetricians and paediatricians, studying the treatment effects in pregnancy via disease‐specific registries can be challenging. Data on icatibant use in pregnant and lactating women remain limited and will continue to be monitored through routine pharmacovigilance.

In total, 11,626 HAE attacks occurred among 722 patients with HAE‐C1INH treated with icatibant. The median (range) time to treatment, time to HAE attack resolution after treatment and total attack duration were 1 (0–95), 6.5 (0–100) and 9.5 (0–100) hours, respectively. The median (range) of injections per attack was 1 (1–5). Among paediatric patients treated with icatibant (35 patients aged < 18 years), all safety and effectiveness observations were similar to the adult population. Sexual hormone laboratory values were not captured during routine visits and therefore it was not possible to characterise the effect of icatibant, if any, on sexual maturation.

The IOS has been pivotal in improving our understanding of healthcare experiences for patients with HAE and informing optimal care. It has also shaped the development of subsequent registries critical for advancing care for rare diseases [8]. Registry data have inherent limitations, as data may be incomplete due to data collection occurring at the physician's discretion during routine care rather than on a study‐specific schedule with defined assessments. However, a strength is the ability to aggregate large amounts of data over time and provide valuable insights into the impact of treatments on patients. Key achievements of the IOS registry include demonstration of icatibant real‐world effectiveness for attack treatment in HAE‐C1INH, 1 dose being effective for most attacks, and early treatment facilitated by self‐administration leading to faster attack resolution [6]. Observations over 15 years in the IOS show icatibant effectiveness consistent with efficacy reported in clinical studies. The safety data align with the established safety profile of icatibant, with no new safety concerns identified during 15 years of follow‐up in routine clinical practice. As such, the data presented here substantiate the positive benefit‐risk profile of icatibant, recommended as an on‐demand treatment option for HAE attacks in the International/Canadian and World Allergy Organization/European Academy of Allergy and Clinical Immunology guidelines [9].

## Author Contributions

All authors were involved in interpreting the results, contributed to the preparation of this manuscript and provided final approval for publication.

## Funding

This study was sponsored by Takeda Development Center Americas Inc., Lexington, MA, USA.

## Conflicts of Interest

Laurence Bouillet has consulted/served as a speaker for, engaged in research and educational projects with, or accepted travel grants from BioCryst, Blueprint Medicines, Celltrion, CSL Behring, GSK, KalVista Pharmaceuticals, Novartis, Otsuka, Pharvaris and Takeda. Werner Aberer has participated in speaker bureaus for BioCryst, CSL Behring, Pharming and Takeda; and has been an advisory board member for Takeda. Teresa Caballero is a member of advisory boards for Astria Therapeutics, BioCryst, CSL Behring, Ionis Pharmaceuticals, KalVista Pharmaceuticals, Novartis, Otsuka, Pharvaris and Takeda; is a member of speaker bureaus for BioCryst, CSL Behring, Novartis and Takeda; has received grants from CSL Behring and Takeda; has received funding to attend conferences/educational events from BioCryst, CSL Behring, Novartis and Takeda; is/has been a clinical trial/registry investigator for BioCryst, BioMarin, CSL Behring, Ionis Pharmaceuticals, KalVista Pharmaceuticals, Novartis, Pharvaris and Takeda; and is a researcher from the IdiPAZ programme for promoting research activities. Anete S. Grumach has received speaker/consultancy fees from AstraZeneca, Astria Therapeutics, BioMarin, CSL Behring, KalVista Pharmaceuticals, MultiCare, Pharvaris, Pint‐Pharma and Takeda; a scholarship from the Brazilian National Center of Research (CNPq); and a grant of researcher initiative from Takeda. Hilary J. Longhurst has received research grant support and/or speaker/consultancy fees from Astria Therapeutics, CSL Behring, Intellia Therapeutics, KalVista Pharmaceuticals, Pharvaris and Takeda. Andrea Zanichelli received speaker/consultancy fees from Astria Therapeutics, BioCryst, CSL Behring, KalVista Pharmaceuticals, Pharming, Pharvaris and Takeda. Irmgard Andresen is an employee of Takeda Pharmaceuticals International AG and holds stock/options in Takeda Pharmaceutical Company Limited. Alissa Dangel is an employee of Takeda Pharmaceuticals USA Inc. and holds stock/options in Takeda Pharmaceutical Company Limited. Natalie Khutoryansky is an employee of Cytel, contracted by Takeda Development Center Americas Inc.

## Data Availability

The dataset, including the redacted study protocol, redacted statistical analysis plan, and individual participants’ data supporting the results reported in this article, will be made available within 3 months from initial request to researchers who provide a methodologically sound proposal. The data will be provided after its de‐identification, in compliance with applicable privacy laws, data protection, and requirements for consent and anonymization.
